# Investigating zinc toxicity responses in marine *Prochlorococcus* and *Synechococcus*

**DOI:** 10.1099/mic.0.001064

**Published:** 2021-06-25

**Authors:** Indrani Sarker, Lisa R. Moore, Sasha G. Tetu

**Affiliations:** ^1^​ Department of Molecular Sciences, Macquarie University, Sydney, Australia; ^2^​ MQ Biomolecular Discovery Research Centre, Macquarie University, Sydney, Australia

**Keywords:** anthropogenic pollution, cell membrane integrity, marine picocyanobacteria, maximum quantum yield of PSII, stress response, zinc toxicity

## Abstract

Marine plastic pollution is a growing concern worldwide and has the potential to impact marine life via leaching of chemicals, with zinc (Zn), a common plastic additive, observed at particularly high levels in plastic leachates in previous studies. At this time, however, little is known regarding how elevated Zn affects key groups of marine primary producers. Marine cyanobacterial genera *Prochlorococcus* and *Synechococcus* are considered to be some of the most abundant oxygenic phototrophs on earth, and together contribute significantly to oceanic primary productivity. Here we set out to investigate how two *Prochlorococcus* (MIT9312 and NATL2A) and two *Synechococcus* (CC9311 and WH8102) strains, representative of diverse ecological niches, respond to exposure to high Zn concentrations. The two genera showed differences in the timing and degree of growth and physiological responses to elevated Zn levels, with *Prochlorococcus* strains showing declines in their growth rate and photophysiology following exposure to 27 µg l^−1^ Zn, while *Synechococcus* CC9311 and WH8102 growth rates declined significantly on exposure to 52 and 152 µg l^−1^ Zn, respectively. Differences were also observed in each strain’s capacity to maintain cell wall integrity on exposure to different levels of Zn. Our results indicate that excess Zn has the potential to pose a challenge to some marine picocyanobacteria and highlights the need to better understand how different marine *Prochlorococcus* and *Synechococcus* strains may respond to increasing concentrations of Zn in some marine regions.

## Introduction

Human activities are increasingly impacting the marine realm, often resulting in detrimental effects on the health and productivity of marine ecosystems [1, 2]. Plastic debris is now recognized as an important anthropogenic pollutant, and plastic litter has now been found in all surveyed marine areas [3, 4]. Plastics commonly found in the ocean can act as a source and vector for various organic pollutants as well as a range of metals, including zinc (Zn) and copper (Cu) [5–7]. Zn is widely used in the manufacture of many plastic and rubber items [8], and leaching of high levels of Zn from both new and aged plastic and car tyre rubber has been reported in multiple studies looking at plastic pollution impacts on marine organisms [5, 9–15]. In addition to plastics, other anthropogenic pollutants including antifouling agents [16] and atmospheric aerosols [17] have been reported as possible sources of increased Zn in the marine environment. Given the increasing likelihood that marine environments will experience elevated Zn levels, there is now a need to consider the effect this may have on key marine organisms.

Zinc homeostasis is critical to many organisms, including microbes, with cells required to maintain minute concentrations for enzymatic function and cell growth whilst avoiding excess accumulation due to the risk of toxicity at higher levels [18–21]. In aquatic microorganisms, Zn toxicity has been shown to affect different cellular processes, but such investigations have primarily focused on freshwater organisms. For example, freshwater green algae exposed to Zn at concentrations ranging from 10 to 100 mg l^−1^ showed growth and productivity inhibition, and induction of oxidative stress and antioxidant activity [22, 23], while freshwater cyanobacteria including *Microcystis, Anabaena, Spirulina* and *Synechococcus* suffered growth inhibition and reduced chlorophyll *a* content following exposure to Zn concentrations ranging from 0.25 to 10 mg l^−1^ [24–29].

Relatively few marine microorganisms have been characterized regarding their Zn toxicity response, as Zn levels in marine waters are typically low. The total dissolved Zn concentration is estimated to be ~0.3 nM in the surface waters of the North Atlantic and North Pacific Oceans [30–32]. The majority of this Zn (~98 %) is bound to uncharacterized organic ligands resulting in very low concentrations (1–20 pM) of free Zn (Zn^2+^) in examined surface waters [31, 33–37]. While such typically low levels mean that most marine organisms will never encounter Zn at toxic levels, the increased burden of anthropogenic pollutants within marine environments suggests investigations of Zn toxicity in these organisms are now timely. Free Zn has been reported at concentrations ranging from 3.27 to 9.66 µg l^−1^ in Port Jackson estuary, Australia [38], 6.50–28 µg l^−1^ in Mersey estuary, UK [39], and as high as 154.71 µg l^−1^ with an average of 32.15 µg l^−1^ in Kochi estuary, India [40]. While open ocean environments are not expected to widely experience Zn at high concentrations, the accumulation of plastic debris within open ocean gyres has the potential to result in some Zn transport, with studies indicating that plastic pollution may travel to these regions relatively rapidly [41] and within the timeframe that additive Zn may still be leaching from plastic debris [15].

The marine microorganisms that have been studied in regard to Zn toxicity responses show similar growth and physiological affects to those seen in freshwater strains, but the Zn levels tolerated are often lower. Growth inhibition has been reported for Zn additions ranging from 10 to 100 µM for marine chlorophytes, coccolithophores and the filamentous cyanobacterium *Oscillatoria* [42], 0.15 mg l^−1^ for brown algae [43], 20–1240 µg l^−1^ for several marine diatoms [40, 44, 45]. For marine picocyanobacteria, there are only a few reports to date looking directly at Zn toxicity. *Synechococcus* sp. (CCMP 1333) was observed to show reduced growth and photosynthetic activity at a total Zn concentration of 10^−5^ M (654 µg l^−1^) [46] while natural populations of marine *Synechococcus* were reported to show low to moderate growth inhibition at ~700 µg l^−1^ Zn [47]. While studies on zinc toxicity are limited, there are a number of studies suggesting that marine picocyanobacteria are sensitive to relatively low levels of other marine pollutants, with *Prochlorococcus* tending to be particularly strongly impacted by such exposures. This includes various organic pollutants such as polycyclic aromatic hydrocarbon (PAHs) [48–50] as well as metal pollutants including Cu [51], cadmium (Cd) and lead (Pb) [52, 53] with different isolates or populations often varying in their specific sensitivity to such toxicants. Our previous work also showed *Prochlorococcus* MIT9312 and NATL2A were negatively affected by exposure to leachates from unweathered and weathered polyvinyl chloride (PVC) matting and high-density polyethylene (HDPE) bags, which comprised a complex mix of organic and inorganic substances, including Zn [14, 15].

Due to their important role in marine ecosystems and their sensitivity to examined anthropogenic stressors, we chose to investigate how marine *Prochlorococcus* and *Synechococcus* isolates are affected by Zn toxicity. The *Prochlorococcus* strains tested were MIT9312, a high-light-adapted strain isolated from the Gulf Stream [54], and *Prochlorococcus* sp. NATL2A, a low-light-adapted ecotype isolated from the North Atlantic Ocean [55], which were the subject of leachate toxicity experiments [14]. Two *Synechococcus* strains, *Synechococcus* sp. CC9311 (clade I), isolated from the edge of the California current [56], and *Synechococcus* sp. WH8102 (clade III), isolated from the oligotrophic southern Sargasso sea [57], were chosen as they were expected to show distinct responses due to the different ocean habitats in which they dominate and past work indicating differences in metal requirements and sensitivities [58, 59]. *In vitro* experiments were conducted to measure growth, photosynthetic parameters and cell membrane integrity following exposure to a range of ZnCl_2_ concentrations, providing information on both physiological and population growth impacts.

## Methods

### Cell culture and growth monitoring

Cyanobacterial strains *Prochlorococcus* MIT9312 and NATL2A and *Synechococcus* CC9311 and WH8102 were investigated for their Zn toxicity response. All cultures were acclimated to AMP1 media for at least three transfers, then under experimental light and temperature conditions for at least three transfers to ensure balanced growth prior to Zn experiments. To facilitate strain comparisons, all cultures were grown in AMP1, a defined artificial seawater medium [60]. Incubations were conducted at 22 °C with shaking at 100 r.p.m. (Infors HT Multitron incubator), with LED cool white irradiance of 40 µmol photons m^−2^ s^−1^ for MIT9312, CC9311 and WH8102 and 20 µmol photons m^−2^ s^−1^ for NATL2A (a lower light level was used for acclimation and experimental testing in this strain as it is a low-light-adapted ecotype). All glassware was acid-washed as previously described [14]. While *Prochlorococcus* and *Synechococcus* cultures were not axenic, staining and flow cytometry was carried out prior to experiments which showed that very low levels of non-photosynthetic bacteria were found relative to the photosynthetic bacteria. Use of AMP1 medium, which contains no added carbon, helps to keep heterotrophic bacteria at low levels, particularly over the short time frames of these experiments. The experiments were set up so that in all cases parameters that are specific to the photosynthetic bacteria were measured and all flow cytometry measurements were made thresholding on chlorophyll and pulse amplitude modified (PAM; for photosynthetic efficiency) fluorescence results looking specifically at photosynthetic parameters.

### Experimental setup

Four independent cultures of each strain from acclimated cultures which were in mid-exponential growth (~5×10^6^ cells ml^−1^) were used as inoculates (10 ml) for the replicates of each Zn treatment experiment in a total volume of 40 ml in a 150 ml glass Erlenmeyer flask. The experimental medium was made by adding ZnCl_2_ to unamended AMP1 [containing 2 µg l^−1^ (0.01 µM) Zn], which was used as the control medium. For *Prochlorococcus,* Zn concentrations were 14.5, 27, 52 and 152 µg l^−1^ (0.1, 0.2, 0.4 and 1.1 µM) total Zn (Zn concentration in AMP1 plus that of added ZnCl_2_ stock), while for *Synechococcus* 27, 52, 152 and 452 µg l^−1^ (0.2, 0.4, 1.1 and 3.3 µM) total Zn was used. The Zn concentrations selected for each strain were based on preliminary experiments, which indicated that the two *Synechococcus* isolates used in this work could tolerate somewhat higher Zn levels than the two *Prochlorococcus* strains. The concentration of Zn in AMP1 media and ZnCl_2_ stocks were confirmed by inductively coupled plasma-mass spectrometry (ICP-MS) analysis at the Environmental Analysis Laboratory (EAL), Southern Cross University (Sydney, Australia) from subsamples collected into acid-washed vials and acidified with 1 % nitric acid. At initiation of the experiment (0 h) the four independent parent cultures were measured to determine population densities, photophysiological health and cell membrane integrity (see below), and then used to inoculate each of the control and ZnCl_2_ treatments. All control and treatment cultures were then subsampled at 3, 24 and 48 h for all parameters, as described below.

### Flow cytometry

The concentration and chlorophyll fluorescence of *Prochlorococcus* and *Synechococcus* populations was quantified using a CytoFLEX S flow cytometer and data were analysed with the CytExpert software (Beckman Coulter). *Prochlorococcus* and *Synechococcus* cells were identified by chlorophyll fluorescence and side angle light scattering properties using blue laser 488 nm excitation and 690 nm emission. Samples were gated so that only cells with chlorophyll fluorescence intensities indicative of healthy cells were counted. At each time point, subsamples were collected from each flask and analysed immediately with the Cytoflex flow cytometer.

### Photosynthetic efficiency measurements

Photosystem II (PSII) quantum yield was measured using a Phyto Pulse Amplitude Modified (PAM) Fluorometer (Walz) as described previously [61]. Briefly, a 2 ml aliquot was placed in a quartz cuvette and dark adapted for 5 min. Basal fluorescence (*F*_0_) was then determined under modulated light (excitation at 440 nm for *Prochlorococcus* cells and 520 nm for *Synechococcus*). After addition of 50 µM of PSII inhibitor 3-(30,4-dichlorophenyl)-1,1dimethylurea (DCMU), maximal fluorescence (*F*_m_) was measured by applying a light saturating pulse. The maximum quantum yield of PSII, *F*_v_/*F*_m_, was calculated as *F*_v_/*F*_m_=[*F*_m_ − *F*_o_]/*F*_m_, where *F*_v_ is variable fluorescence. For each individual biological replicate three technical replicates were measured at each time point.

### Cell membrane integrity determination (SYTOX assay)

The membrane integrity of *Prochlorococcus* and *Synechococcus* cells was measured on fresh samples within 1 h of collection using the ‘live/dead’ SYTOX Green stain (# S7020; Invitrogen) and detected with flow cytometry, based on previously reported methods [62] with slight modifications optimized for our cells, as previously detailed [15]. Healthy *Prochlorococcus* cultures treated with 1 % paraformaldehyde and *Synechococcus* cultures treated with heat (60 °C for 60 min at 350 r.p.m.) served as dead controls and were used to determine the green fluorescence level (emission at 525/40 nm) of SYTOX positive stained cells (i.e. those with damaged or compromised membranes). The proportion of SYTOX-stained cells within the total chlorophyll fluorescence population (*Prochlorococcus*) and total phycoerythrin fluorescence population (*Synechococcus*) was calculated for each sample within each treatment.

### Statistical analysis

The growth, photophysiology and SYTOX data were analysed using one-way ANOVA followed by Tukey’s multiple comparison test with 95 % confidence interval using GraphPad Prism 8 for Windows (GraphPad Software). All data from Zn treatments were compared to the AMP1 control at the matching timepoint and considered significant (*) at *P*<0.01. The growth rate was calculated by fitting linear regression models to the flow cytometric population density measurements from 3 to 48 h. Concentration response curves and IC50 values for all four strain were analysed by fitting the non-linear regression model (least squares regression) using GraphPad Prism 8 software.

## Results

### Growth rate analysis

**Fig. 1.**
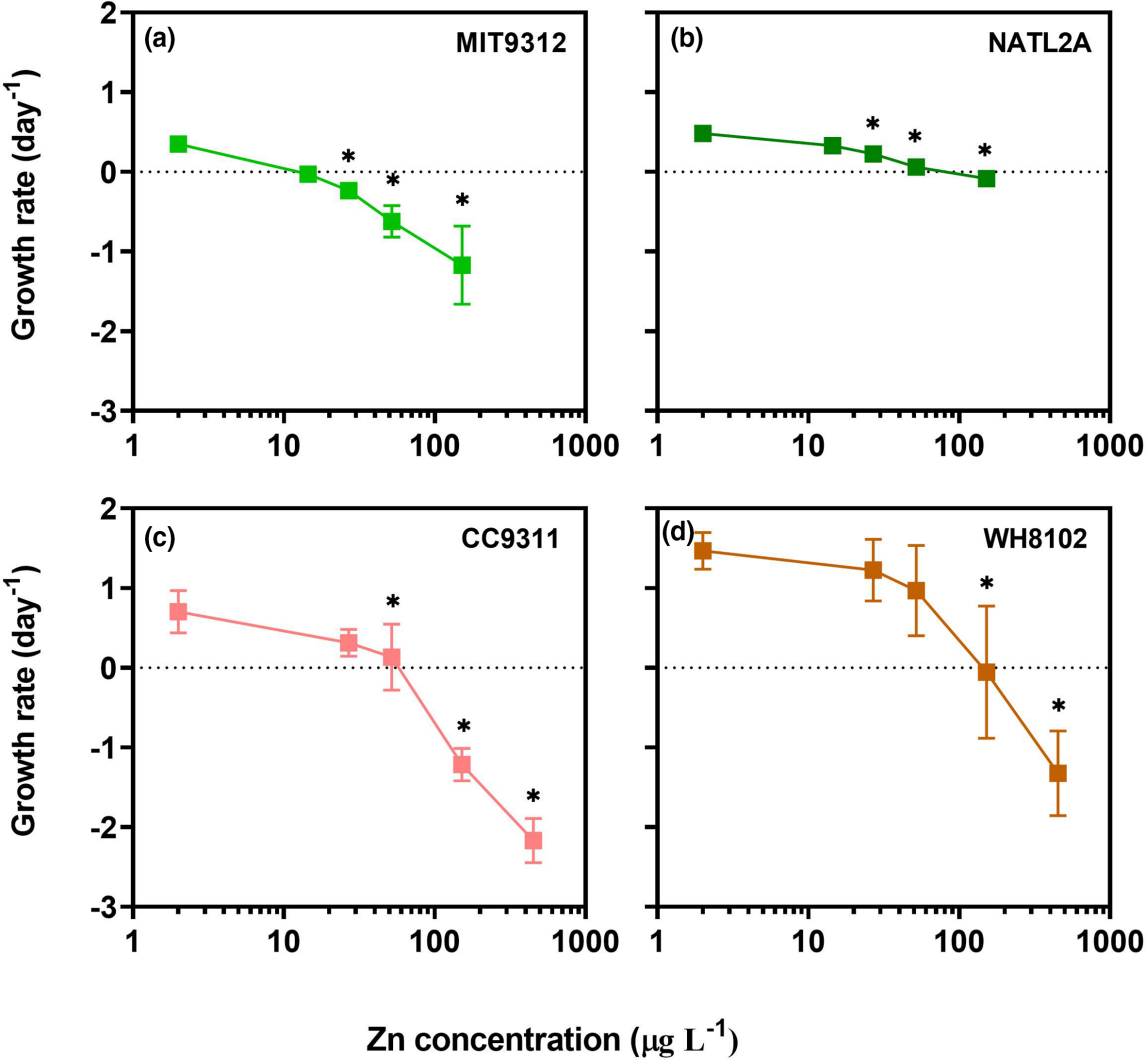
Growth rate of *Prochlorococcus* strains (**a**) MIT9312 and (**b**) NATL2A and *Synechococcus* strains (**c**) CC9311 and (**d**) WH8102 in AMP1 artificial seawater medium with a range of zinc (Zn) concentrations. Experiments were conducted over 48 h using four independent biological replicates for each strain and condition. Growth rates were determined based on flow cytometric cell counts taken from 3 to 48 h after experimental set up. All data are represented as mean values of all replicate cultures with error bars indicating the standard deviation (error bars not visible where values are smaller than symbols). Asterisks (*) indicate that the growth rate was significantly (*P*<0.01) different for a specific Zn treatment concentration relative to the control (AMP1 containing 2 µg l^−1^ Zn) (P values are provided in Table S1).

To investigate how exposure to a range of Zn concentration affects marine *Prochlorococcus* and *Synechococcus*, ZnCl_2_ was added to artificial seawater media at a range of concentrations prior to inoculation with healthy cultures of *Prochlorococcus* MIT9312 and NATL2A and *Synechococcus* CC9311 and WH8102. For all tested *Prochlorococcus* and *Synechococcus* strains, negative growth rates were observed for treatments with Zn concentrations of 152 µg l^−1^ and above (Fig. 1). Each strain, however, showed distinct differences in their precise growth rate response to each of the different Zn additions (Fig. 1). Both *Prochlorococcus* MIT9312 and NATL2A showed significant reductions (*P*<0.01) in growth rates on exposure to 27 µg l^−1^ total Zn, a concentration that did not significantly impair growth in the two tested *Synechococcus* strains. The growth rate reductions for *Prochlorococcus* MIT9312 were more marked than for NATL2A across the full range of concentrations tested (Fig. 1a, b). In *Synechocococus* significant reductions (*P*<0.01) in growth were seen at Zn concentrations ≥52 µg l^−1^ for CC9311 while in WH8102 significant growth rate declines occurred at ≥152 µg l^−1^ Zn (Fig. 1c, d). Responses between genera also varied in that *Prochlorococcus* strains maintained a steady decline in growth rate in response to Zn stress up to 152 µg l^−1^ (the highest Zn concentration measured), whereas *Synechococcus* strains showed a more marked decline in growth rate following exposure to Zn concentrations starting at 52 µg l^−1^ (Fig. 1).

### Photophysiological activity

**Fig. 2.**
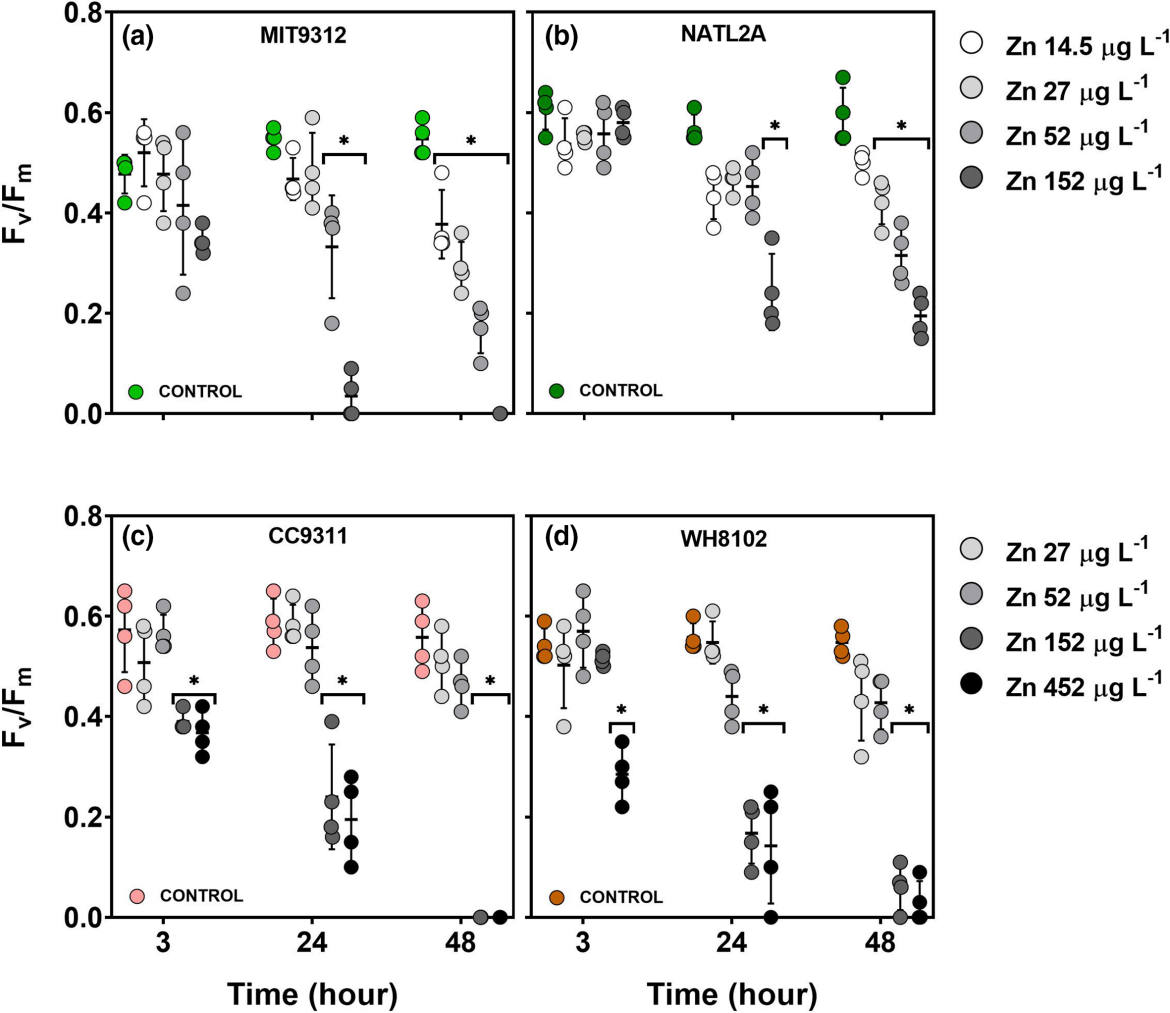
Maximum quantum yield of photosystem II (*F*_v_/*F*_m_) for *Prochlorococcus* MIT9312 (**a**), *Prochlorococcus* NATL2A (**b**), *Synechococcus* CC9311 (**c**) and *Synechococcus* WH8102 (**d**) exposed to different concentrations of zinc (Zn) for different exposure times. The AMP1 control Zn concentration was 2 µg l^−1^ for all four strains. Experiments were conducted over 48 h using biological replicates (*n*=4) for all. Symbols show the value for each biological replicate, with the bar indicating the mean±sd for each treatment at each time point. Asterisks (*) signify treatments for which measurements were significantly (*P*<0.01) different compared to the control (AMP1 containing 2 µg l^−1^ Zn) at the examined time point (P values are provided in Table S2).

The maximum quantum yield of PSII (*F*_v_/*F*_m_) was measured to determine how excess Zn affects *Prochlorococcus* and *Synechococcus* photophysiology. As was observed with growth rate, Zn exposure affected *F*_v_/*F*_m_ in a concentration-dependent manner, and strain-specific differences were observed with respect to concentration sensitivity and the timing of responses (Fig. 2). Both *Prochlorococcus* strains showed a delayed photophysiological response to Zn exposures relative to the two *Synechococcus* strains. Significant reductions (*P*<0.01) in *F*_v_/*F*_m_ were observed starting at 24 h for *Prochlorococcus* MIT9312 for treatments with Zn concentrations of 52 µg l^−1^ and for all concentrations tested (14.5–152 µg l^−1^) by 48 h (Fig. 2a). For *Prochlorococcus* NATL2A, significant reductions were reported for only 152 µg l^−1^ Zn at 24 h and for 27 µg l^−1^ and higher concentrations at the 48 h time point, indicating lower sensitivity than MIT9312 (Fig. 2b). *Synechococcus* strains showed a more rapid response to high levels of Zn with significant (*P*<0.01) reductions in *F*_v_/*F*_m_ observed following 3 h of exposure to Zn concentrations of 152 µg l^−1^ and above in CC9311 (Fig. 2c) and 452 µg l^−1^ in WH8102 (Fig. 2d). *Synechococcus* WH8102 showed a significant decline in *F*_v_/*F*_m_ for 152 µg l^−1^ Zn by 24 h, but neither *Synechococcus* strain showed significant photophysiological impairment at lower concentrations throughout the 48 h experiment.

**Fig. 3.**
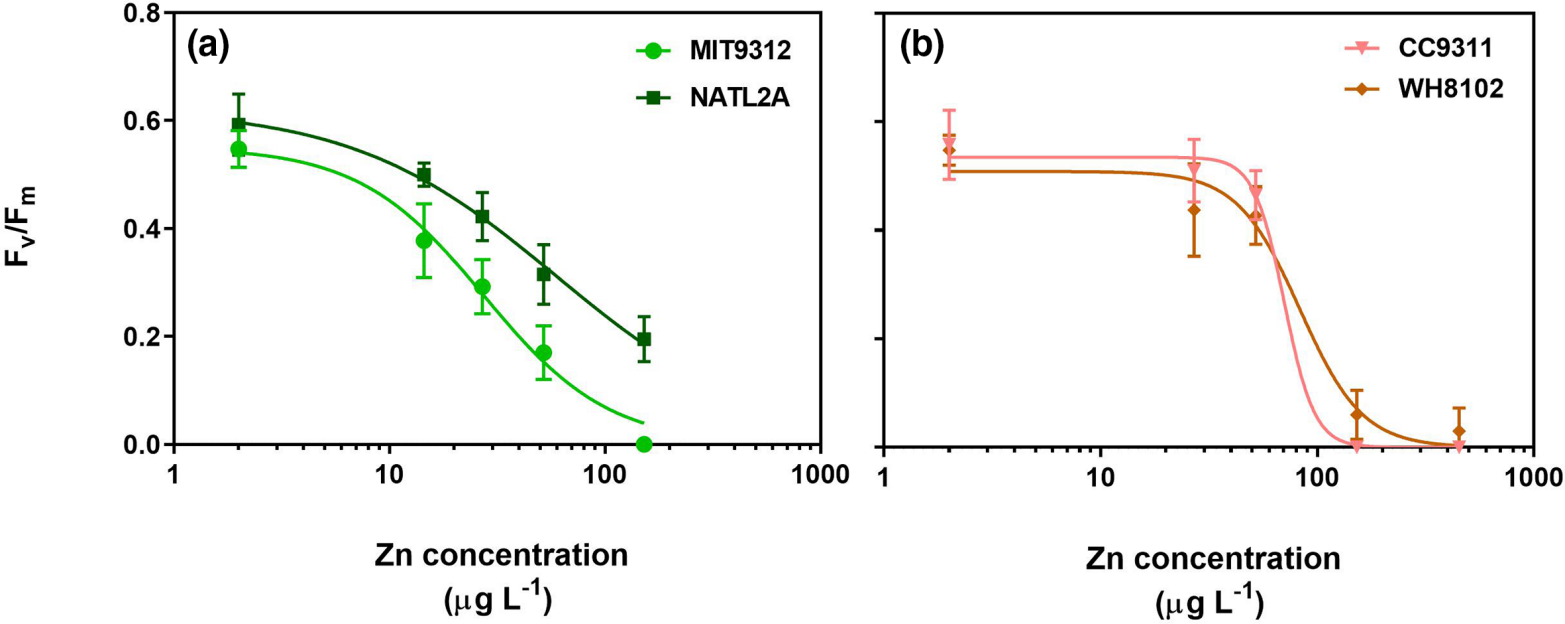
Concentration–response curve derived from non-linear regression analysis, showing inhibition of maximum quantum yield of photosystem II (*F*_v_/*F*_m_) of four marine cyanobacterial strains: (**a**) *Prochlorococcus* MIT9312 and NATL2A, and (**b**) *Synechococcus* CC9311 and WH8102 after 48 h of exposure to a range of zinc (Zn) concentrations (plotted as total Zn, calculated from the concentration of Zn in AMP1 plus added ZnCl_2_).

Photophysiological responses in each strain after 48 h of exposure to each Zn concentration were used to generate concentration–response curves to facilitate strain sensitivity comparisons (Fig. 3). Differences were observed in the shape of concentration–response curves between *Prochlorococcus* (Fig. 3a) and *Synechococcus* (Fig. 3b) species. *Prochlorococcus* strains showed a steady decline in their *F*_v_/*F*_m_ in response to increasing Zn concentrations, while *F*_v_/*F*_m_ for *Synechococcus* declined sharply with exposures above ~52 µg l^−1^ Zn. Comparing *Prochlorococcus* strains also indicated some difference in sensitivity, with NATL2A *F*_v_/*F*_m_ less affected for each Zn concentration than MIT9312. The IC50 (concentration at which *F*_v_/*F*_m_ is reduced by 50 %) values for each of these strains was determined from these plotted curves (Table 1). *Prochlorococcus* MIT9312 had the lowest IC50 at 28±3 µg l^−1^ while for NATL2A the IC50 was 60±10 µg l^−1^. The two *Synechococcus* strains were found to be more tolerant than *Prochlorococcus* and showed less difference in their response to the tested Zn concentrations, with IC50 values of 70±16 and 83±10 µg l^−1^ for strain CC9311 and WH8102, respectively.

**Table 1.** The zinc (Zn) concentration determined to cause 50 % inhibition (IC50) in *F*_v_/*F*_m_ of *Prochlorococcus* and *Synechococcus* strains after 48 h of exposure, based on non-linear regression fit analysis

Cyanobacterial strain	IC50	IC50 se	d.f.	*R* ^2^
	(µg l^−1^)			
MIT9312	28	3	17	0.9
NATL2A	60	10	17	0.9
CC9311	70	16	17	1.0
WH8102	83	10	17	0.9

### Cell membrane integrity

**Fig. 4.**
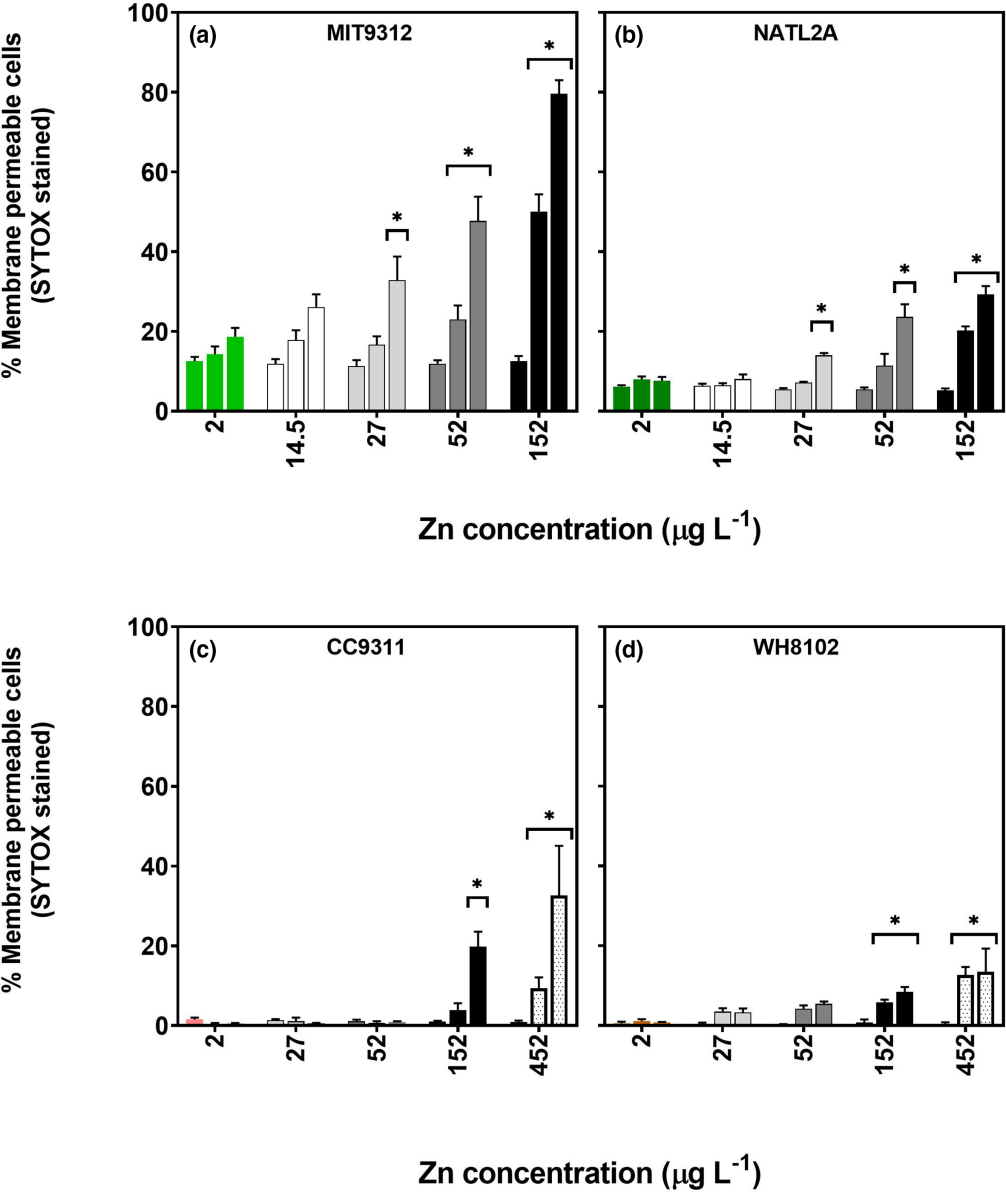
Proportions of cell populations stained with SYTOX Green (i.e. membrane compromised) after exposure to different concentrations of zinc (Zn) in *Prochlorococcus* MIT9312 (**a**) and NATL2A (**b**) and *Synechococcus* CC9311 (**c**) and WH8102 (**d**). The three grouped bars for each Zn concentration represent different time points (3, 24 and 48 h from left to right) for all figures. Column height is the mean value of the biological replicates (*n*=4) for each time point and Zn concentration, with error bars representing the standard deviation. Asterisks (*) signify data points where measurements were significantly (*P*<0.01) different compared to the control (AMP1 containing 2 µg l^−1^ Zn) at each time point (P values are provided in Table S3).

To determine whether Zn exposure results in cell membrane damage, the percentage of each cell population with compromised membranes was determined for each strain using the ‘live/dead’ SYTOX Green stain at all time points and for all Zn concentrations. The tested *Prochlorococcus* and *Synechococcus* strains all exhibited concentration-dependent membrane damage but differed in the Zn concentration and time after exposure at which membrane damage manifested, as well as in the proportion of the population showing membrane damage (Fig. 4a–d). *Prochlorococcus* strains did not show significant changes in membrane integrity at 3 h for any of the Zn concentrations (Fig. 4a, b). By 24 h, *Prochlorococcus* MIT9312 populations showed a significant (*P*<0.01) increase in membrane compromised cells for Zn concentrations of 52 and 152 µg l^−1^ and for 27–152 µg l^−1^ by 48 h (Fig. 4a). For *Prochlorococcus* NATL2A, significant proportions of membrane damaged cells were only observed for Zn concentrations of 152 µg ^−1^ at 24 h, and 27–152 µg l^−1^ at the 48 h time point (Fig. 4b). In contrast, *Synechococcus* strains showed a later and less severe response to the higher Zn concentrations with significant (*P*<0.01) increases in the proportion of membrane damaged cells only observed at 24 h of exposure to 152 µg l^−1^ for CC9311 and 48 h for WH8102 (Fig. 4c, d). The highest concentration tested, 452 µg l^−1^ Zn, resulted in significant (*P*<0.01) membrane damage at 24 and 48 h for both CC9311 and WH8102 (Fig. 4c, d).

## Discussion

Understanding how marine microbes respond to anthropogenic stressors, such as exposure to high levels of heavy metals, is becoming more important as pressures on the marine environment continue to grow. In cyanobacteria, the ability to handle metal toxicity has been shown to be achieved by triggering various defence mechanisms, such as organic or inorganic precipitation, biotransformation, active transport and sequestration (in extracellular exopolysaccharides and/or intracellular polyphosphate granules, or metal-binding proteins such as metallothionein) [63]. For well-studied freshwater cyanobacteria, such defensive mechanisms have been shown to assist with tolerating relatively high concentrations of Zn pollution [26]. However, there is a limit to the Zn concentrations which can be tolerated, above which cyanobacterial defensive mechanisms become unable to neutralize excess Zn, and negative effects are seen in terms of growth and a range of other physiological parameters. The threshold concentrations tolerated by different freshwater cyanobacteria have been shown to vary considerably. For example, *Microcystis aeruginosa* can tolerate up to 0.25 mg l^−1^ of ZnCl_2_ for more than 2 weeks in polluted aquatic environments [28] or in culture [64] while freshwater *Synechococcus* sp. IU 625 is highly tolerant to Zn and can survive up to 25 mg l^−1^ ZnCl_2_ over 29 days [27]. While less is known about Zn toxicity tolerance in marine cyanobacteria, one study looking at natural populations of marine *Synechococcus* [47] observed 50 % growth rate inhibition following 72 h of exposure to 713 µg l^−1^ Zn, which falls within the range of Zn levels found to show negative effects in some freshwater cyanobacteria. Our study using laboratory cultures of *Prochlorococcus* and marine *Synechococcus* showed more severe growth inhibition after only 48 h of exposure with Zn concentrations of 27–152 µg l^−1^, depending on the strain. This suggests that there may be substantial variability in terms of the mechanisms that marine cyanobacteria have available to defend against excess Zn, and certain strains may lack some or most of the systems for reducing excess Zn observed for freshwater strains.

In our study, *Synechococcus* strains showed a greater capacity to tolerate excess Zn than the *Prochlorococcus* strains, which are smaller in terms of both genome size and cell size. Both *Prochlorococcus* strains showed significant impacts on growth, photophysiology and membrane integrity following exposure to 27 µg l^−1^ Zn, while this concentration did not significantly affect either *Synechococcus* strain. In order to deal with excess Zn, marine cyanobacteria have been shown to rely on a mechanism of Zn sequestration by bacterial metallothioneins (BmtAs, SmtAs) [65], which are small cytosolic proteins rich in cysteine residues that bind and sequester metal ions, thereby preventing deleterious interactions [20]. Genome analyses have shown that *Synechococcus* strains CC9311 and WH8102 both encode a gene for metallothionein BmtA, which may provide them with some capacity to withstand elevated Zn levels, while *Prochlorococcus* MIT9312 and NATL2A genomes lack a metallothionein encoding gene, and it is not known what mechanisms *Prochlorococcus* have for dealing with excess Zn [21, 66]. Our findings are largely consistent with past work on marine cyanobacterial tolerance to other metals and stressors, with previous work indicating that *Prochlorococcus* species tend to be more sensitive than *Synechococcus* to Cu [51], cadmium and lead [52], and PAHs [67]. Differences in average cell size have previously been put forward as a major factor contributing to varied sensitivity to organic pollutants and metals, both for laboratory cultures and for natural communities of *Prochlorococcus* and *Synechococcus* [49, 52]. Smaller cells have been suggested to have a higher capacity to incorporate contaminants, due to their larger surface area to volume ratio [68, 69]. The smaller cell size of *Prochlorococcus* could potentially contribute to higher sensitivity to Zn compared to the *Synechococcus*, although further investigation is needed to confirm this.

Strain-specific differences were also observed, with *Prochlorococcus* MIT9312, a high-light-adapted strain, being particularly sensitive to Zn toxicity and showing more severe impacts for all tested growth and physiology parameters compared to NATL2A, a low-light-adapted strain. This differs to what has been seen in work on Cu toxicity tolerance using different representative *Prochlorococcus* isolates (low-light SS120 and MIT9313; high-light MED4, MIT9311 and MIT9401), where members of the high-light-adapted clade were observed to tolerate higher levels of Cu than strains belonging to the low-light-adapted group [51]. However, this difference may relate to the specific strains used in our study, as past work suggests NATL2A may be atypical of low-light strains in its capacity to respond to changing conditions, such as high light [70] and phosphorus limitation [71]. NATL2A also encodes an unusually high number of genes encoding high-light inducible proteins (HLIPs) compared to other *Prochlorococcus* low-light ecotypes, and these genes have been shown to be transcriptionally responsive to a number of stress conditions and may provide photoprotection [72], so their abundance in this strain may contribute to stress tolerance. Our finding that NATL2A can tolerate a greater range of Zn concentrations than MIT9312 is consistent with what we previously observed in terms of responses to plastic leachate, containing a mix of Zn and other inorganic and organic substances, which also showed NATL2A to be more tolerant of this toxicant [14].

The two tested *Synechococcus* strains also differed in their responses to Zn. Strain WH8102 was able to retain a positive growth rate and maintain maximum photosynthetic quantum yield for a greater range of Zn concentrations than CC9311, while the membrane damage was essentially the same between the two strains, except at the 48 h time point for the two highest concentrations (152 and 452 µg l^−1^) where CC9311 showed significantly (*P*<0.05, Student’s *t*-test) greater damage. Previous genome analyses of picocyanobacteria have noted that marine *Synechococcus* strain CC9311 encoded more genes involved in metal homeostasis as well as metal enzymes and sensor kinases compared to strain WH8102 [58]. Growth assays showed coastal *Synechococcus* strain CC9311 exhibits higher tolerance to Cu shock than open ocean strain WH8102, as well as differentially transcribing a much higher proportion of the genome under this stress (29.2 % in CC9311 compared to 5.5 % in WH8102) [59]. It is possible that different picocyanobacteria strains may differ in their sensitivity to specific metals, as work by Debelius and colleagues which looked at both Zn and Cu sensitivity found that *Synechococcus* surface populations from the Mediterranean Sea were more sensitive to Zn than Atlantic Ocean populations, while Cu sensitivities were less variable between these two in terms of their chlorophyll *a* signals [47]. Although CC9311 may be less sensitive to Cu toxicity than WH8102, our study indicates that it is more sensitive to Zn toxicity than WH8102, implying that these two *Synechococcus* strains have different mechanisms of homeostasis/toxicity to the two metals.

Our study revealed that the photophysiology of all cyanobacterial strains is strongly affected in response to Zn toxicity, with significant inhibition of maximum photosynthetic quantum yield (*F*_v_/*F*_m_) observed in all four strains within 24 h of exposure. Zn has previously been found to affect photosynthetic efficiency of marine *Synechococcus* sp. CCMP1333 [46]. Previous work investigating Zn toxicity mechanisms in *Synechocystis* indicated that high Zn interferes with electron transfer at the reducing side of the PSII reaction centre and may also affect energy transfer among antenna pigments [73]. In plant chloroplasts it has been shown that inhibition of PSII activity is linked to Zn dissociating oxygen-evolving polypeptides from PSII and oxygen-evolving complex (OEC) [74]. Although none of these processes have been directly investigated in oligotrophic marine picocyanobacteria, these represent potential mechanisms by which Zn may exert the photosynthetic effects we observed in this study. Another study which focused on cobalt (Co) limitation in *Prochlorococcus* MIT9215 found that Zn toxicity could occur due to its competitive inhibition with Co uptake by the periplasmic manganese (Mn) soluble binding protein MntC for the MntABC transport system [75]. All the strains in our study do have genes for MntC, thus making this another possible mechanism of Zn toxicity for marine picocyanobacteria. Direct genome-wide transcriptomic or proteomic investigations into Zn toxicity responses in these strains would help to determine how excess Zn affects physiological processes, particularly photosynthesis.

## Conclusions

Our study reveals how *Prochlorococcus* and *Synechococcus* growth, photophysiology and membrane integrity are affected by exposure to a range of Zn concentrations. Marine cyanobacteria are important contributors to marine primary productivity but have not been widely examined with regard to their sensitivity to anthropogenic pollutants, such as Zn. Here we show 27 µg l^−1^ ZnCl_2_ substantially reduced growth, photosynthetic efficiency and cell membrane integrity of *Prochlorococcus* strains, while both *Synechococcus* strains were affected by concentrations of 152 µg l^−1^ and above, indicating that marine picocyanobacteria may have limited mechanisms to cope with Zn toxicity, compared to freshwater cyanobacteria. Differences in the timing and degree of growth and physiological responses between the tested strains indicate that environmental Zn toxicity could have varied impacts on closely related marine picocyanobacteria, potentially due to variations in the mechanisms available to cope with excess Zn. As anthropogenic inputs of Zn into the marine environment appear to be growing, this *in vitro* study provides an important preliminary step in understanding how these ecologically significant photosynthetic bacteria may respond to exposure to increased Zn.

## Supplementary Data

Supplementary material 1
