# Supplementary material for: Investigating zinc toxicity responses in marine Prochlorococcus and Synechococcus
Source: Microbiology (Reading). 2021 Jun 25;167(6):001064. doi: 10.1099/mic.0.001064 (PMC8374608; doi:10.1099/mic.0.001064)
Supplement: Supplementary material 1 [file mic-167-1064-s001.pdf]

Title: Investigating zinc toxicity responses in marine *Prochlorococcus* and *Synechococcus*

Supplementary material

Supplementary Table 1: Results of one-way ANOVA followed by Tukey's multiple comparison tests for growth rate (Figure 1). MIT = MIT9312; NAT = NATL2A; control = 2µg L^{-1} total Zn.

**Comparisons**	**P Value**	**Comparisons**	**P Value**
**Fig. 1A**		**Fig. 1B**	
MIT control vs. Zn 14.5 µg L^{-1}	0.0123	NAT control vs. Zn 14.5 µg L^{-1}	0.0154
MIT control vs. Zn 27 µg L^{-1}	0.0002	NAT control vs. Zn 27 µg L^{-1}	0.0002
MIT control vs. Zn 52 µg L^{-1}	<0.0001	NAT control vs. Zn 52 µg L^{-1}	<0.0001
MIT control vs. Zn 152 µg L^{-1}	<0.0001	NAT control vs. Zn 152 µg L^{-1}	<0.0001
Zn 14.5 µg L^{-1} vs. Zn 27 µg L^{-1}	0.2737	Zn 14.5 µg L^{-1} vs. Zn 27 µg L^{-1}	0.1574
Zn 14.5 µg L^{-1} vs. Zn 52 µg L^{-1}	0.0002	Zn 14.5 µg L^{-1} vs. Zn 52 µg L^{-1}	<0.0001
Zn 14.5 µg L^{-1} vs. Zn 152 µg L^{-1}	<0.0001	Zn 14.5 µg L^{-1} vs. Zn 152 µg L^{-1}	<0.0001
Zn 27 µg L^{-1} vs. Zn 52 µg L^{-1}	0.0099	Zn 27 µg L^{-1} vs. Zn 52 µg L^{-1}	0.0090
Zn 27 µg L^{-1} vs. Zn 152 µg L^{-1}	<0.0001	Zn 27 µg L^{-1} vs. Zn 152 µg L^{-1}	<0.0001
Zn 52 µg L^{-1} vs. Zn 152 µg L^{-1}	0.0005	Zn 52 µg L^{-1} vs. Zn 152 µg L^{-1}	0.0230
**Fig. 1C**		**Fig. 1D**	
CC9311 control vs. Zn 27 µg L^{-1}	0.0386	WH8102 control vs. Zn 27 µg L^{-1}	0.9057
CC9311 control vs. Zn 52 µg L^{-1}	0.0022	WH8102 control vs. Zn 52 µg L^{-1}	0.414.59
CC9311 control vs. Zn 152 µg L^{-1}	<0.0001	WH8102 control vs. Zn 152 µg L^{-1}	0.0006
CC9311 control vs. Zn 452 µg L^{-1}	<0.0001	WH8102 control vs. Zn 452 µg L^{-1}	<0.0001
Zn 27 µg L^{-1} vs. Zn 52 µg L^{-1}	0.5852	Zn 27 µg L^{-1} vs. Zn 52 µg L^{-1}	0.8909
Zn 27 µg L^{-1} vs. Zn 152 µg L^{-1}	<0.0001	Zn 27 µg L^{-1} vs. Zn 152 µg L^{-1}	0.0030
Zn 27 µg L^{-1} vs. Zn 452 µg L^{-1}	<0.0001	Zn 27 µg L^{-1} vs. Zn 452 µg L^{-1}	<0.0001
Zn 52 µg L^{-1} vs. Zn 152 µg L^{-1}	<0.0001	Zn 52 µg L^{-1} vs. Zn 152 µg L^{-1}	0.0171
Zn 52 µg L^{-1} vs. Zn 452 µg L^{-1}	<0.0001	Zn 52 µg L^{-1} vs. Zn 452 µg L^{-1}	<0.0001
Zn 152 µg L^{-1} vs. Zn 452 µg L^{-1}	<0.0001	Zn 152 µg L^{-1} vs. Zn 452 µg L^{-1}	0.0032

Supplementary Table 2: Results of one-way ANOVA followed by Tukey's multiple comparison tests for maximum photosynthetic quantum yield F_v/F_m (Figure 2). MIT = MIT9312; NAT = NATL2A; control = 2 µg L^{-1} total Zn.

Comparisons	P Value		
**Fig. 2A**	**3 hour**	**24 hour**	**48 hour**
MIT control vs. Zn 14.5 µg L^{-1}	0.9376	0.4271	0.0009
MIT control vs. Zn 27 µg L^{-1}	>0.9999	0.6165	<0.0001
MIT control vs. Zn 52 µg L^{-1}	0.7936	0.0021	<0.0001
MIT control vs. Zn 152 µg L^{-1}	0.1757	<0.0001	<0.0001
Zn 14.5 µg L^{-1} vs. Zn 27 µg L^{-1}	0.9376	0.9971	0.1235
Zn 14.5 µg L^{-1} vs. Zn 52 µg L^{-1}	0.3672	0.0611	0.0001
Zn 14.5 µg L^{-1} vs. Zn 152 µg L^{-1}	0.0456	<0.0001	<0.0001
Zn 27 µg L^{-1} vs. Zn 52 µg L^{-1}	0.7936	0.0329	0.0148
Zn 27 µg L^{-1} vs. Zn 152 µg L^{-1}	0.1757	<0.0001	<0.0001
Zn 52 µg L^{-1} vs. Zn 152 µg L^{-1}	0.7210	<0.0001	0.0009
**Fig. 2B**			
NAT control vs. Zn 14.5 µg L^{-1}	0.2137	0.0194	0.0771
NAT control vs. Zn 27 µg L^{-1}	0.4341	0.0805	0.0008
NAT control vs. Zn 52 µg L^{-1}	0.5277	0.0426	<0.0001
NAT control vs. Zn 152 µg L^{-1}	0.9156	<0.0001	<0.0001
Zn 14.5 µg L^{-1} vs. Zn 27 µg L^{-1}	0.9860	0.9385	0.1716
Zn 14.5 µg L^{-1} vs. Zn 52 µg L^{-1}	0.9604	0.9932	0.0003
Zn 14.5 µg L^{-1} vs. Zn 152 µg L^{-1}	0.6259	0.0006	<0.0001
Zn 27 µg L^{-1} vs. Zn 52 µg L^{-1}	0.9998	0.9966	0.0326
Zn 27 µg L^{-1} vs. Zn 152 µg L^{-1}	0.8856	0.0002	<0.0001
Zn 52 µg L^{-1} vs. Zn 152 µg L^{-1}	0.9406	0.0003	0.0156

**Fig. 2C**			
CC9311 control vs. Zn 27 µg L^{-1}	0.5341	>0.9999	0.5516
CC9311 control vs. Zn 52 µg L^{-1}	0.9997	0.8870	0.0580
CC9311 control vs. Zn 152 µg L^{-1}	0.0039	<0.0001	<0.0001
CC9311 control vs. Zn 452 µg L^{-1}	0.0014	<0.0001	<0.0001
Zn 27 µg L^{-1} vs. Zn 52 µg L^{-1}	0.6411	0.8870	0.5995
Zn 27 µg L^{-1} vs. Zn 152 µg L^{-1}	0.0779	<0.0001	<0.0001
Zn 27 µg L^{-1} vs. Zn 452 µg L^{-1}	0.0282	<0.0001	<0.0001
Zn 52 µg L^{-1} vs. Zn 152 µg L^{-1}	0.0055	0.0003	<0.0001
Zn 52 µg L^{-1} vs. Zn 452 µg L^{-1}	0.0019	<0.0001	<0.0001
Zn 152 µg L^{-1} vs. Zn 452 µg L^{-1}	0.9809	0.9048	>0.9999
**Fig. 2D**			
WH8102 control vs. Zn 27 µg L^{-1}	0.8625	0.9995	0.0753
WH8102 control vs. Zn 52 µg L^{-1}	0.9600	0.1457	0.0466
WH8102 control vs. Zn 152 µg L^{-1}	0.9600	<0.0001	<0.0001
WH8102 control vs. Zn 452 µg L^{-1}	0.0001	<0.0001	<0.0001
Zn 27 µg L^{-1} vs. Zn 52 µg L^{-1}	0.4939	0.2059	0.9989
Zn 27 µg L^{-1} vs. Zn 152 µg L^{-1}	0.9979	<0.0001	<0.0001
Zn 27 µg L^{-1} vs. Zn 452 µg L^{-1}	0.0007	<0.0001	<0.0001
Zn 52 µg L^{-1} vs. Zn 152 µg L^{-1}	0.6721	0.0003	<0.0001
Zn 52 µg L^{-1} vs. Zn 452 µg L^{-1}	<0.0001	0.0001	<0.0001
Zn 152 µg L^{-1} vs. Zn 452 µg L^{-1}	0.0004	0.9828	0.9321

Supplementary Table 3: Results of one-way ANOVA followed by Tukey's multiple comparison tests for cell membrane integrity/Sytox assay (Figure 4). MIT = MIT9312; NAT = NATL2A; control = 2 μg L^{-1} total Zn.

Comparisons	P Value		
**Fig. 4A**	**3 hour**	**24 hour**	**48 hour**
MIT control vs. Zn 14.5 μg L^{-1}	0.9169	0.4782	0.1744
MIT control vs. Zn 27 μg L^{-1}	0.5861	0.7849	0.0031
MIT control vs. Zn 52 μg L^{-1}	0.9393	0.0072	<0.0001
MIT control vs. Zn 152 μg L^{-1}	>0.9999	<0.0001	<0.0001
Zn 14.5 μg L^{-1} vs. Zn 27 μg L^{-1}	0.9629	0.9822	0.2486
Zn 14.5 μg L^{-1} vs. Zn 52 μg L^{-1}	>0.9999	0.1611	<0.0001
Zn 14.5 μg L^{-1} vs. Zn 152 μg L^{-1}	0.9393	<0.0001	<0.0001
Zn 27 μg L^{-1} vs. Zn 52 μg L^{-1}	0.9461	0.0630	0.0022
Zn 27 μg L^{-1} vs. Zn 152 μg L^{-1}	0.6302	<0.0001	<0.0001
Zn 52 μg L^{-1} vs. Zn 152 μg L^{-1}	0.9576	<0.0001	<0.0001
**Fig. 4B**			
NAT control vs. Zn 14.5 μg L^{-1}	0.9223	0.6299	0.9982
NAT control vs. Zn 27 μg L^{-1}	0.2123	0.9393	0.0015
NAT control vs. Zn 52 μg L^{-1}	0.1553	0.0328	<0.0001
NAT control vs. Zn 152 μg L^{-1}	0.0365	<0.0001	<0.0001
Zn 14.5 μg L^{-1} vs. Zn 27 μg L^{-1}	0.0516	0.9628	0.0027
Zn 14.5 μg L^{-1} vs. Zn 52 μg L^{-1}	0.0359	0.0021	<0.0001
Zn 14.5 μg L^{-1} vs. Zn 152 μg L^{-1}	0.0076	<0.0001	<0.0001
Zn 27 μg L^{-1} vs. Zn 52 μg L^{-1}	0.9997	0.0076	<0.0001
Zn 27 μg L^{-1} vs. Zn 152 μg L^{-1}	0.8520	<0.0001	<0.0001
Zn 52 μg L^{-1} vs. Zn 152 μg L^{-1}	0.9249	<0.0001	0.0039
**Fig. 4C**			
CC9311 control vs. Zn 27 μg L^{-1}	0.9439	0.9785	>0.9999
CC9311 control vs. Zn 52 μg L^{-1}	0.3954	0.9996	>0.9999

CC9311 control vs. Zn 152 µg L^{-1}	0.2924	0.0432	0.0024
CC9311 control vs. Zn 452 µg L^{-1}	0.1155	<0.0001	<0.0001
Zn 27 µg L^{-1} vs. Zn 52 µg L^{-1}	0.8101	0.9960	>0.9999
Zn 27 µg L^{-1} vs. Zn 152 µg L^{-1}	0.6911	0.1198	0.0024
Zn 27 µg L^{-1} vs. Zn 452 µg L^{-1}	0.3654	<0.0001	<0.0001
Zn 52 µg L^{-1} vs. Zn 152 µg L^{-1}	0.9994	0.0629	0.0028
Zn 52 µg L^{-1} vs. Zn 452 µg L^{-1}	0.9271	<0.0001	<0.0001
Zn 152 µg L^{-1} vs. Zn 452 µg L^{-1}	0.9762	0.0011	0.0487
**Fig. 4D**			
WH8102 control vs. Zn 27 µg L^{-1}	0.9895	0.0614	0.6917
WH8102 control vs. Zn 52 µg L^{-1}	0.7469	0.0104	0.1594
WH8102 control vs. Zn 152 µg L^{-1}	0.9779	0.0003	0.0081
WH8102 control vs. Zn 452 µg L^{-1}	0.9923	<0.0001	<0.0001
Zn 27 µg L^{-1} vs. Zn 52 µg L^{-1}	0.9424	0.8811	0.7896
Zn 27 µg L^{-1} vs. Zn 152 µg L^{-1}	0.8370	0.0719	0.0955
Zn 27 µg L^{-1} vs. Zn 452 µg L^{-1}	>0.9999	<0.0001	0.0006
Zn 52 µg L^{-1} vs. Zn 152 µg L^{-1}	0.4217	0.3298	0.5167
Zn 52 µg L^{-1} vs. Zn 452 µg L^{-1}	0.9322	<0.0001	0.0055
Zn 152 µg L^{-1} vs. Zn 452 µg L^{-1}	0.8537	<0.0001	0.1147
